# Annotations

**Published:** 1887-07-30

**Authors:** 


### Annotations.

**Wonderful Cure! Physic, Faith, or Neither?**

At the British Home for Incurables, an "incurable" has lately been the subject of a "cure" which would have made the reputation of a faith-healer and the fortune of a "quack" doctor. Hannah D——, aged 37, was elected an in-patient under two medical certificates. One stated that she was speechless in consequence of an obscure brain disease; the other that she had a tumour of the brain, causing paralysis of the muscles of speech. The two certificates represented two types of the genus doctor, the cautious and the confident. But the doctors were not alone in their judgment. The woman had been under a throat specialist at the throat department of a large hospital, and from this she was discharged as incurable from paralysis. The cause of these serious symptoms was apparently a nervous shock produced by the removal of eighteen teeth at two sittings Her voice left her at once after this mild attack of dentistry, and for a year and a half she could only speak in whispers. Since that time she had been unable to utter a sound of any kind, felt continual pain at the top of the head, and could not walk about from giddiness—to use her own expression, "reeling about as if she were tipsy." What a charming array of symptoms for a "quack" advertisement! On June 9th of this year Dr. Rugg and Dr. Ferrier, of vivisection fame, saw her together at the hospital and made a thorough examination. They concluded that there was no tumour of the brain, and indeed no organic disease at all. They electrified the windpipe, but could elicit no sound. Still convinced that there was no definite disease, they decided upon leaving the patient and her incurable disease entirely alone. For six days this original treatment was pursued, and the doctor never went near her. As the advertisements say, "the result was truly marvellous"; and as orthodoxy repeats, "the medicine acted like a charm." On the sixth day she suddenly found her lost speech. After trying to converse with the patients for some time, she all at once exclaimed, "I think I have got my speech." Sure enough she had. She now talks freely; says she is quite well; has a good appetite and good spirits, and takes the air daily on Clapham Common. Will this case teach the faith-healers or the believers in quack remedies anything? We fear not.

**Tweedledum or Tweedledee?**

A boy Main, who appears to have been suffering from disease of bone in the legs, was some time ago placed in the Launceston Union. Not making a rapid recovery there, he was passed on to the Rowe Dispensary. When he was admitted some of the Dispensary officials seem to have made public complaint that the boy had been sent from the Union in a dirty, filthy, and uncared-for condition. Naturally the Union authorities were up in arms in defence of their officials, the parish doctor and the workhouse matron. The committee of the Rowe Dispensary seem to have taken very high ground in the matter. They have not only declined to meet the guardians, but have apparently placed hindrances in the way of an official inquiry by the conjoint bodies. The guardians have therefore held an inquiry of their own, and all the witnesses examined being on one side, have had no difficulty in coming to the conclusion that the Dispensary committee are entirely wrong in their facts, and absolutely unpardonable in their manners. The Rowe people say the boy was dirty, and having said that, they will not budge one way or the other. The guardians say he was not dirty,—and there the matter stands. Was he dirty or was he not? Are the Dispensary people unmannerly or are they not? These are knotty questions, and perhaps the world at Rowe and outside it will never know the truth At any rate, we hope that poor Main is now having his legs well washed and nice soft poultices applied, with plenty of clean linen rag to use when necessary That, after all, to make a very obvious pun, is for him the "main" point. We would advise the guardians and the Infirmary committee to dine together over the business. Life is too short for quarrelling about trifles.

**Women and Work.**

Mr. Walter Besant, whose letter we publish elsewhere, is going to try to reduce some of his ideals to practice. Under his auspices, and those of the Hon. M. Stanley, Frederick Verney, Esq., William Hill, Esq., Athro A. Knight, Esq., and others, it is proposed to "hold a conference towards the end of the year, at which the facts of the female labour-market may be clearly and dispassionately set forth; the extent, area, and nature of the evils which undoubtedly exist, may be laid down with some precision; and, if that may also follow, remedies or alleviation may be found." Mr. Walter Besant and his friends are doing right to try to find some remedies for evils which are great, terrible, and practically unchecked. On the one hand there is the grim science pointing out to us that human nature is such that it will always try its utmost to "buy in the cheapest market and sell in the dearest." On the other hand is the pity born of natural gentleness, education, and religious feeling. The one is a monstrous giant which fills the world. The other is —what? A mere voice pleading for pity and help for those who have no weapons of self-defence. Gibbon tells us that "persuasion is the resource of the feeble"; and he adds, what we might have expected him to add—"but the feeble can seldom persuade." With our whole heart we go with Mr. Besant and his friends; but, with a fear which springs from remembered experience, we anticipate an up-hill fight which may weary the stoutest hearts and paralyse the most resolute hands. The mere attempt, however, deserves, and will ensure, the warmest sympathy of every man and woman who has an educated conscience and a true human soul.

